# Investigation of the Electrochemical Behavior of CuO-NiO-Co_3_O_4_ Nanocomposites for Enhanced Supercapacitor Applications

**DOI:** 10.3390/ma17163976

**Published:** 2024-08-10

**Authors:** Karthik Kannan, Karuppaiya Chinnaiah, Krishnamoorthy Gurushankar, Raman Krishnamoorthi, Yong-Song Chen, Paskalis Sahaya Murphin Kumar, Yuan-Yao Li

**Affiliations:** 1 Department of Mechanical Engineering, Advanced Institute of Manufacturing with High-Tech Innovations, National Chung Cheng University, Chia-Yi 621301, Taiwan; karthikkannanphotochem@gmail.com; 2 Multifunctional Laboratory, International Research Centre, Kalasalingam Academy of Research and Education, Krishnankoil, Virudhunagar 626126, Tamil Nadu, India; yasaganchinna38@gmail.com (K.C.); gurushankar01051987@gmail.com (K.G.); 3 Laboratory of Computational Modelling Drugs, Higher Medical and Biological School, South Ural State University, 454080 Chelyabinsk, Russia; 4 Pharamaceutics Laboratory, Graduate Institute of National Products, Chang Gung University, Kweishan, Taoyuan 33305, Taiwan; krishmicro3@gmail.com; 5 Department of Chemical Engineering, National Chung Cheng University, Chia-Yi 621301, Taiwan; marpinindian@gmail.com (P.S.M.K.); chmyyl@ccu.edu.tw (Y.-Y.L.); 6 Advanced Institute of Manufacturing with High-Tech Innovations, National Chung Cheng University, Chia-Yi 621301, Taiwan

**Keywords:** metal oxide composites, morphological, electrochemical, supercapacitors

## Abstract

In the present study, composites incorporating NiO-Co_3_O_4_ (NC) and CuO-NiO-Co_3_O_4_ (CNC) as active electrode materials were produced through the hydrothermal method and their performance was investigated systematically. The composition, formation, and nanocomposite structure of the fabricated material were characterized by XRD, FTIR, and UV–Vis. The FE-SEM analysis revealed the presence of rod and spherical mixed morphologies. The prepared NC and CNC samples were utilized as supercapacitor electrodes, demonstrating specific capacitances of 262 Fg^−1^ at a current density of 1 Ag^−1^. Interestingly, the CNC composite displayed a notable long-term cyclic stability 84.9%, which was observed even after 5000 charge–discharge cycles. The exceptional electrochemical properties observed can be accredited to the harmonious effects of copper oxide addition, the hollow structure, and various metal oxides. This approach holds promise for the development of supercapacitor electrodes. These findings collectively indicate that the hydrothermally synthesized NC and CNC nanocomposites exhibit potential as high-performance electrodes for supercapacitor applications.

## 1. Introduction

Recent developments in electrochemical devices have created significant new avenues for investigating the properties of various metal oxides and their composites [1]. These advancements have led to noteworthy improvements in the performance of electrical energy storage devices, particularly in attractive characteristics like energy density, cycle life, and power density [2]. These enhancements have the potential to broaden the applications of electrochemical capacitors across various sectors in multiple domains such as electronics, transportation, industrial processes, and healthcare [3]. Supercapacitors have become essential in meeting the growing energy storage needs of renewable energy systems. The goal of ongoing research on supercapacitors is to increase their adaptability so that they can be widely used in a variety of usual applications. This includes electric vehicles, electronic devices, and power grids, highlighting the crucial importance of supercapacitors in the modern world [4]. In general, supercapacitors store the energy through two different mechanisms: pseudocapacitors and electrical double-layer capacitors [5]. These mechanisms are characterized by rapid faradaic redox reactions and charge separation at the interface between the electrode and electrolyte. The high specific capacitance of precious transition metal oxides, such as RuO_2_ and IrO_2_, made them popular as electrode materials at first, but their cost and toxicity constrain their practical use [6].

Consequently, there is a demand for cost-efficient and environmentally friendly transition metal oxides (TMOs) such as CuO, Co_3_O_4_, NiO, MnO_2_, MoS_2_, and ZnO/MnOx, which exhibit diverse structures and morphologies [7,8]. Notably, double TMOs, such as oxides of Cu, Ni, and Co and oxides of Zn and V, have attracted noteworthy interest due to the occurrence of ions from two distinct metals and the resulting synergistic effects between these elements [9]. These compounds provide a larger number of active sites and display superior electrical conductivity compared to individual metal oxides (e.g., Co, Mn, Mo, Ni, Cu, and Sn, etc.) [10]. It is important to note that these mixed metal oxides possess notable characteristics, including natural abundance, good electrical conductivity, and cost-effectiveness [11].

Among TMOs, CuO is a well-known p-type semiconductor with a narrow bandgap. Their remarkable stability, durability, and excellent electrochemical properties make CuO nanostructures ideal candidates for use as electrode materials in electrochemical energy storage applications [12]. NiO, on the other hand, is a p-type semiconductor with a unique electronic structure that supports reversible Faradaic redox reactions at the electrode–electrolyte interface, with a theoretical capacitance of 2573 F/g. The abundance of nickel enhances the scalability and market potential of NiO-based supercapacitors. Environmentally and safety-wise, NiO is a preferable electrode material, as it is non-toxic and environmentally friendly [13].

Co_3_O_4_ is a distinct mixed-valence oxide made up of CoO and Co_2_O_3_. It exhibits a mixed valence state, enabling reversible redox reactions and boasting a high theoretical specific capacitance of 3560 F/g. During the charging and discharging processes, it undergoes reversible redox reactions that facilitate the movement of multiple electrons, enhancing the energy retention capabilities of supercapacitors. It also demonstrates excellent cycle stability, which contributes to a longer lifespan for supercapacitors—an important factor for industrial applications where reliability is crucial. Additionally, its high-rate capability allows for rapid energy storage and release [14].

The Ni-Mn oxide electrode demonstrates a specific capacitance value of 1227 Fg^−1^ while functioning at a current density 10 Ag^−1^. This oxide is synthesized using the chemical bath deposition method at low temperatures. Remarkably, it maintains a capacity retention rate (76.7%) even after undergoing 10^3^ cycles [15]. The research conducted by Askari et al. focused on the utilization of a NiO-Co_3_O_4_-rGO nanocomposite as an electrode for supercapacitors. The results showed that this nanocomposite exhibited a specific capacity of 149 mAh g^−1^ (equivalent to 894 F g^−1^) at a current density 0.5 Ag^−1^. Furthermore, it demonstrated an impressive stability of 95% even after undergoing 6000 cycles. Additionally, the study revealed that NiO-Co_3_O_4_-rGO achieved current densities at 15 and 10 mA cm^−2^ in methanol and ethanol oxidation reactions, respectively [16]. The authors highlighted the superior efficiency of NiO-Co_3_O_4_-rGO as a nanocatalyst for ultra-level nitrite detection compared to NiO-Co_3_O_4_, attributing this to the notable electrical conductivity of rGO and the expansive active surface area of reduced graphene nanosheets [17]. Iqbal et al. successfully produced the binary nanocomposite WS_2_@PANI through hydrothermal and physical mixing techniques. They achieved a specific capacitance over 335 F g^−1^ at 10 mVs^−1^, energy of 80 Wh kg^−1^, and a power density of 800 W kg^−1^ for this material [18]. In another investigation, Iqbal et al. demonstrated the utility of a composite material (MoS_2_@cellulose) made using a microwave-assisted method as an electrode material for supercapacitors [19]. The hydrothermal method offers several advantages over other synthesis techniques for mixed metal oxide composites, including its simplicity and less complicated post-synthetic treatments compared to those of other solution-based methods. Chebrolu et al. [20] investigated various dual transition metal oxides (TMOs) such as Zn-Fe, Zn-Ni, and Zn-Pb oxides, determining that Zn–Ni oxide composites possess high cyclic stability and specific capacities. Zn–Al oxide nanorods displayed a specific capacity of 463.7 Fg^−1^ and outstanding cyclic stability with a retention rate of 96.9% [21].

This present research involves the synthesis of CNC nanocomposites using a hydrothermal method. The resulting composites are analyzed using different characterization techniques, including structural, morphological, elemental, and optical assessments. These composites are evaluated as electrode materials for supercapacitors and demonstrate outstanding electrochemical performance. The remarkable specific capacitance, cyclic stability, and coulombic efficiency of the composites can be linked to their distinctive structural and morphological characteristics.

## 2. Experimental Section

### 2.1. Synthesis of the NiO-Co_3_O_4_ Heterojunction Nanocomposite

The hydrothermal synthesis method was used to produce the NiO-Co_3_O_4_ nanocomposite. A solution containing metal ions (0.5 M Ni(NO_3_)_2_·6H_2_O, Co(NO_3_)_2_·6H_2_O in 40 mL deionized water) and 1 M NaOH solution were mixed for 3 h to obtain a homogeneous liquid. This liquid was then transferred to an autoclave container and heated at 130 °C for 12 h. Following natural cooling to room temperature, the sample was cleaned several times with DI water and subsequently with ethanol. Afterwards, the sample was dried at 100 °C for 12 h and then calcined at 400 °C for 4 h.

### 2.2. Fabrication of the CuO-NiO-Co_3_O_4_ Heterojunction Nanocomposite

**Scheme 1:**
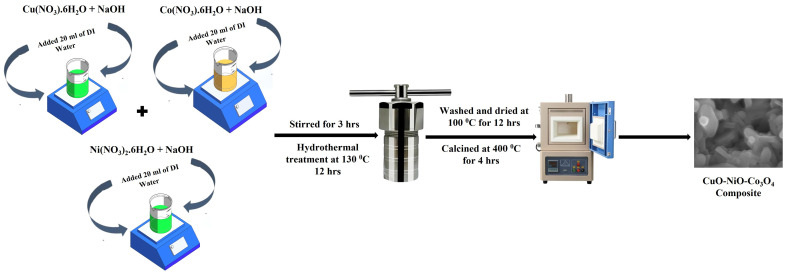
Schematic representation of hydrothermally prepared CNC nanocomposite.

The CuO-NiO-Co_3_O_4_ nanocomposite was synthesized using the hydrothermal method. Metal ions (0.5 M Cu(NO_3_)_2_·6H_2_O, Ni(NO_3_)_2_·6H_2_O, Co(NO_3_)_2_·6H_2_O in 60 mL deionized water) were mixed with 1.5 M NaOH solution for 3 h to form a homogeneous liquid, which was then transferred to an autoclave followed by heating at 130 °C for 12 h. Following natural cooling to room temperature, the sample was cleaned several times with DI water and subsequently with ethanol. Finally, the prepared sample was dried at 100°C for 12 h and calcined at 400 °C for 4 h (Scheme 1). The materials, characterization, and electrochemical setup are shown in the Supplementary Materials.

## 3. Results and Discussion

### 3.1. XRD Analysis

**Figure 1:**
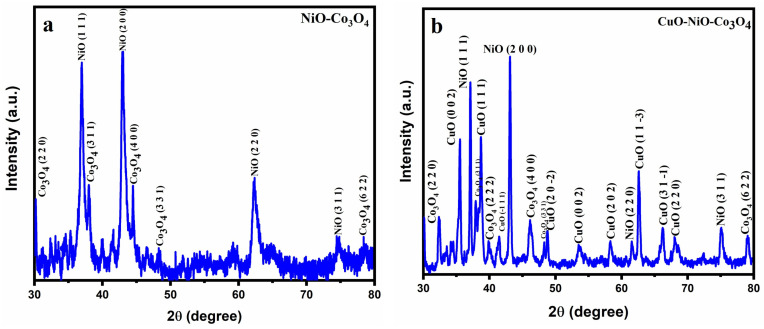
XRD pattern of hydrothermally synthesized (**a**) NC and (**b**) CNC nanocomposites.

The XRD pattern result of the NC and CNC nanocomposites is presented in Figure 1a,b. The observed XRD peaks of CuO, especially at angles of 32.36, 37.04, 38.72, 46.14, and 79.20°, can be attributed to the crystal planes (200), (311), (222), (400), and (622) of CuO (JCPDS no. 74-2120), respectively. Similarly, the peaks noticed at 37.10, 43.12, 62.54, and 75.08° for NiO correspond to the crystal planes (111), (200), (220), and (311) of NiO (JCPDS no. 89-7130). Furthermore, peaks observed at 35.56, 38.72, 48.26, 53.54, 58.30, 62.62, 66.26, and 68.04° are associated with the crystal planes (002), (111), (20-2), (020), (021), (11-3), (31-1), and (220) of CuO (JCPDS no. 48-1548). Notably, the mixed metal nanocomposite’s XRD pattern displayed no impurity peaks, signifying its exceptional purity level. Furthermore, the lattice parameters and crystallite size of the nanocomposites were calculated by the Debye–Scherrer equation [22,23,24,25], as described in Table S1.

### 3.2. Morphological Analysis

**Figure 2:**
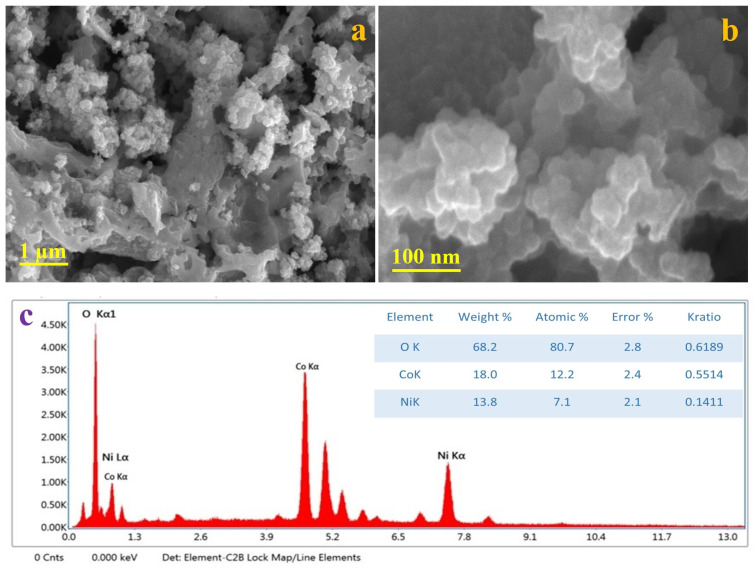
(**a**,**b**) FE-SEM images of the CNC composite with high and low magnifications, (**c**) EDAX spectrum with composition analysis table of the NC nanocomposite.

**Figure 3:**
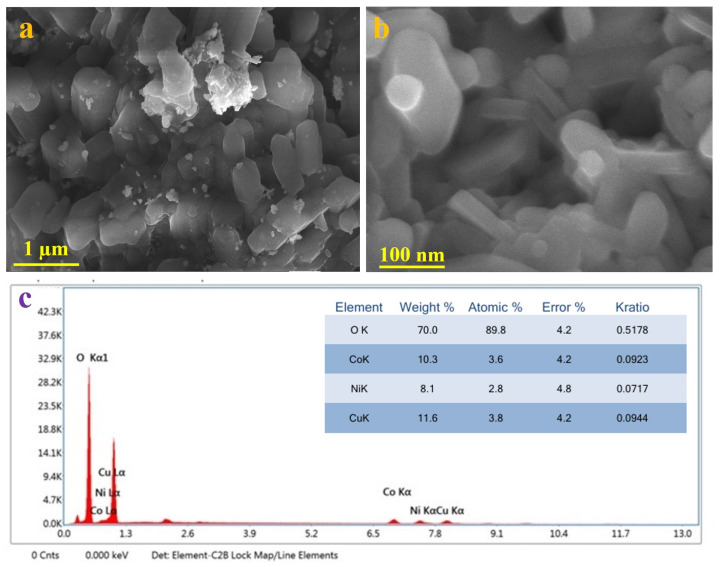
(**a**,**b**) FE-SEM images of the CNC composite with high and low magnifications, (**c**) EDAX spectrum with composition analysis table of the CNC nanocomposite.

**Figure 4:**
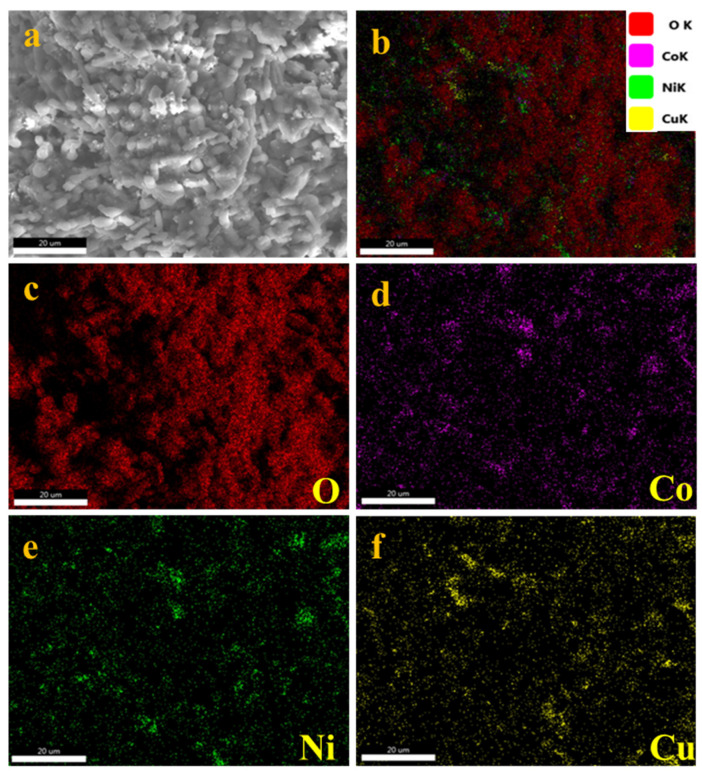
EDS mapping of the CNC composite’s overall spectrum: (**a**,**b**), oxygen (**c**), cobalt (**d**), nickel (**e**), and copper (**f**), respectively.

The FESEM images depicted in Figure 2a,b reveal that the NC composite exhibited spherical and rod-shaped structures. The CNC composite displayed clustered spherical, rod, and tetrahedron-shaped structures (Figure 3a,b). The FE-SEM observations indicate that the clustered surface is planar, featuring cavities and pores that enhance the adsorbent’s surface area [26]. EDAX analysis was directed to determine the composition of the NC and CNC composites, as presented in Figure 2c and Figure 3c. The element maps in Figure 4a–f display the distribution of the EDS elements Cu, Co, Ni, and O, showcasing the successful formation of the composite material. The elemental mapping analysis of the NC composite is presented in Figure S1. FTIR analysis of the prepared CNC composite is provided in Figure S2.

### 3.3. Optical Analysis

**Figure 5:**
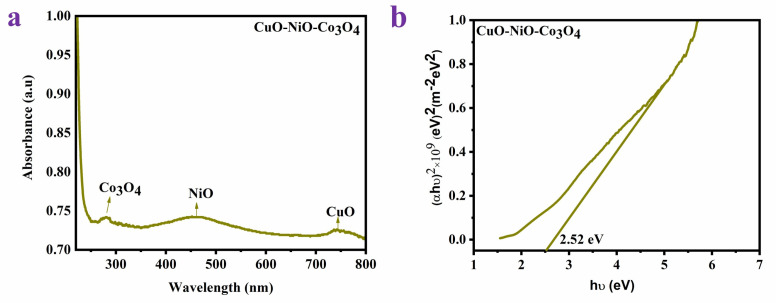
UV–vis absorbance spectrum (**a**) and (**b**) optical bandgap of the prepared CNC nanocomposite.

The optical absorption characteristics of the CNC composite are illustrated in Figure 5a. The absorbance spectrum shows two broad bands, the first ranging from 250 to 280 nm and containing the charge transfer transitions O^2−^ → Co^2+^ and O^2−^ → Co^3+^ and Co(III) at an octahedral site: ^1^A_1g_ → ^1^T_2g_ [27]. Moreover, the octahedrally coordinated Cu^2+^ species is observed at 700−800 nm, which is attributed to the d–d transition band. The absorption edge was observed within the wavelength range of 300–450 nm, with the optical peak occurring at approximately 450 nm. The absorption peak of NiO demonstrates a blue shift attributed to alterations in surface effects, quantum confinement of particles, and crystallite size [28,29,30]. The UV–Vis analysis of the NC composite is provided in Figure S3. The optical bandgap of the fabricated composite can be calculated using Tauc’s relationship. The optical bandgap of the composite can be assessed by plotting (αhν)^2^ against hν. The intersection of the tangent line with the x-axis would give a good approximation of the bandgap for the composite sample (2.52 eV), as shown in Figure 5b.

### 3.4. Electrochemical Studies

#### 3.4.1. CV Analysis

**Figure 6:**
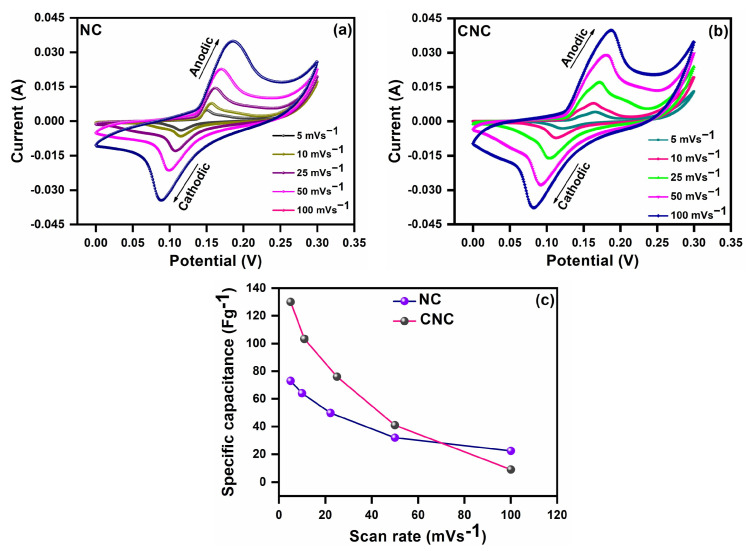
CV curves of (**a**) NC, (**b**) CNC composites, (**c**) calculated specific capacitance variation against the scan rates for NC and CNC electrodes.

The CV analysis of NC and CNC is shown in Figure 6a,b. Measurements were performed with scan rates of 5, 10, 25, 50, and 100 mVs^−1^. Both NC and CNC curves display the pseudocapacitive profiles with non-rectangular shapes, which are adapted to the profiles of intercalation with partial redox. Generally, intercalation, intercalation with partial redox, and the pseudocapacitors’ surface redox profiles are possible occurrences in supercapacitors depending on which materials are used as the electrode. As in the case of non-carbon electrodes, anodic and cathodic peaks existed by oxidation and reduction processes during the electrochemical reactions. Each CV curve exhibits distinct oxidation peaks around 0.17 V and reduction peaks around −0.09 V vs. Ag/AgCl. These cathodic and anodic peaks were shifted towards the negative and positive potentials as the scan rates increased from 5–100 mVs^−1^ by the polarization of active materials (NC and CNC) at higher scan rates. However, the shape of the CV curves does not change entire scan rates indicate that electrodes have good electrochemical stability and reversibility, also indicating an intercalation/surface diffusion-controlled process during the redox process [31]. Hence, the possible reversible redox mechanism occurring in NC and CNC in the KOH electrolyte is represented by Equation (1)

*xH*^+^ + *xe*^−^ + *CNC* ⇄ *H_x_CuONiOCo_3_O_4_* (1)

(*CNC*)*_surface_* + *xH*^+^ + *xe*^−^ ⇄ (*CuONiOCo_3_O_4_* − *H_x_*^+^)*_surface_* (2)

The oxidation and reduction peaks were attributed to the mixed valence state of metal ions (Ni^2+^/Ni^3+^, Cu^+^/Cu^2+^, Co^2+^/Co^3+^). An integrated analysis of the CV curves was utilized to determine the specific capacitances. According to Equation (3), the specific capacitance was calculated by the area under the CV curves.

(3) $$C_{sp}\;\left(CV\right)=\frac{\int Idv}{mv∆V}$$

where “m” is mass of the active material, “I” is the applied current, v-potential window, and “ΔV” is the applied scan rate. Estimated specific capacitance corresponds to the scan rates provided in Figure 6c, i.e., 130, 105, 76, 41, and 17 Fg^−1^ for 5, 10, 25, 50, and 100 mVs^−1^, respectively, for CNC and 73, 64, 49, 32, and 22 Fg^−1^ for NC. High specific capacitance resulted in a low scan rate and rapidly decreased during the changes towards higher scan rate. At lower scan rates, a notable electrochemical performance of the CNC nanocomposite is attributed to the high contact area of the active material, which facilitates the fast ion diffusion and electron transfer at the electrode/electrolyte interface through the synergistic effect of CuO, NiO, and Co_3_O_4_ [32]. Therefore, performance variations of the NC and CNC electrodes were further examined through their profiles.

**Figure 7:**
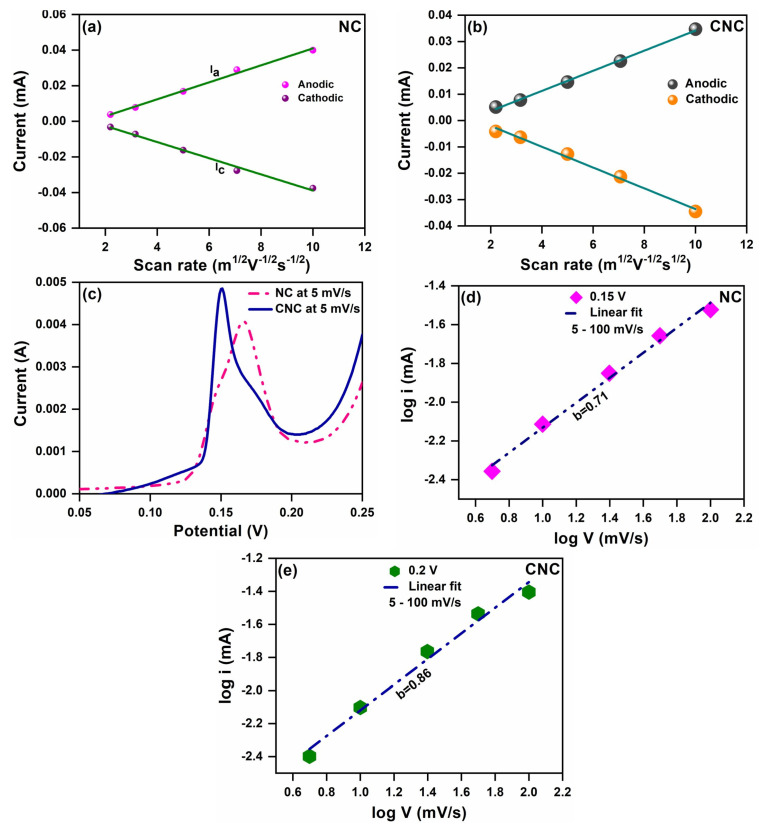
Redox peak relationships: (**a**) NC, (**b**) CNC composites, (**c**) magnitude variation of current in CV curves, (**d**,**e**) determined b-values for NC and CNC composites.

**Figure 8:**
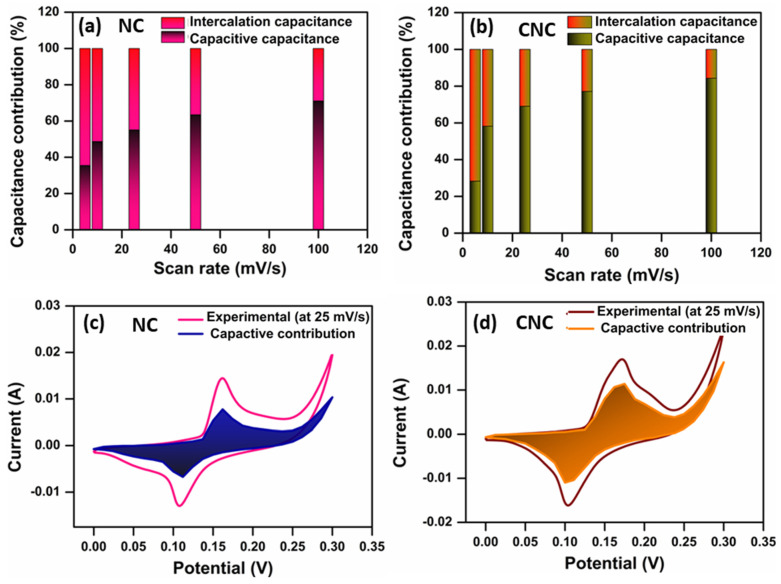
Specific capacitance contributions versus scan rates (**a**) NC, (**b**) CNC electrodes, (**c**,**d**) Resultant specific capacitances at a scan rate of 25 mVs^−1^ for NC and CNC.

The square root of the scan rate against the anodic and cathodic peaks of CV curves is shown in Figure 7a,b. It illustrates the linear relationship of redox peaks for NC and CNC. Hence, it was well-adopted to perform the lower and higher scan rates. The magnitude variations of the current between NC and CNC at 5 mVs^−1^ are noticed in Figure 7c. From these, it is observed that a higher magnitude (blue line) resulted for the CNC electrode. Hence, a more enclosed area was also obtained compared with NC for the CV curves, which was subjected to the active surface area of the electrode and directly involved in the determination of specific capacitance. This result may be due to the higher ionic conductivity of the electrode by the incorporation of Cu^2+^ ions. Hence, the obtained specific capacitance results were evaluated further using the power law as expressed in Equation (4).

(4) $$i=av^{b}$$

where i is the measured current at a given potential V, ν is the scan rate, and a and b are the variable parameters. The b value is calculated from log v vs. log i as shown in Figure 7d,e. The calculated b value of NC and CNC is 0.69 and 0.86, respectively. This result also supported the greater capacitive behavior found for the CNC electrode. In general, if the b-value is 1, it is ideal for capacitance. However, in the present study, the variation of b values was observed, revealing the contribution of capacitance between NC and CNC to its storage mechanism and providing evidence of the mechanism of intercalation/surface diffusion. It was analyzed through Equation (5)

(5) $$\frac{I(V)}{v^{1/2}}=k_{1}v^{\frac{1}{2}}+k_{2}v^{\frac{1}{2}}$$

where k_1_ν and k_2_ν are the current contributions of the capacitance and intercalation of ions. Resultant capacitance contributed by the kinetics of the intercalation of ions can be observed in Figure 8a,b, which is dominated by the lower scan rates. Figure 8c,d show the contribution of the estimated capacitive and intercalation capacitances that resulted in specific capacitances at a scan rate of 25 mVs^−1^. Both electrodes, NC and CNC, have high capacitive capacitance, which is higher in CNC than in NC. The results suggested that the CNC electrode exhibited higher ionic conductivity and superior electrochemical performance due to the fast and active surface diffusion at the interface of the electrode and electrolyte by the enhanced ionic conductivity of Cu^2+^ ions in the CNC nanocomposite electrode. Moreover, CNCs exhibit mixed morphologies such as cubic shape, rod-shaped, and tetrahedron-shaped, which enhance electrode performance for supercapacitors. Recently, Nigam et al. reported the advantages of electrodes with mixed morphologies [33]. A significant amount of Co_3_O_4_ with nanotube morphology in the CNCs facilitates the absorption and retention of electrolyte ions during redox reactions due to its highly active surfaces. The nanorod morphologies of NiO also increase the active sites of the electrode material, albeit to a lesser extent. Additionally, the tetrahedral morphology promotes the growth of other nanoparticles (NiO and Co_3_O_4_) as a nanocomposite, enhancing their conductivity. Consequently, ions can easily penetrate the cavities and pores of the CNCs, improving their electrochemical performance. These morphological features offer benefits for supercapacitor performance beyond those reported for similar transition metal oxide electrodes [34]. This CNC nanocomposite achieves diverse morphologies, notable active surface areas, and specific capacitance, addressing the conductivity issues that have been a drawback of transition metal oxide electrodes. This advancement is crucial for achieving the energy density required for practical applications.

#### 3.4.2. Galvanostatic Charge–Discharge Analysis

**Figure 9:**
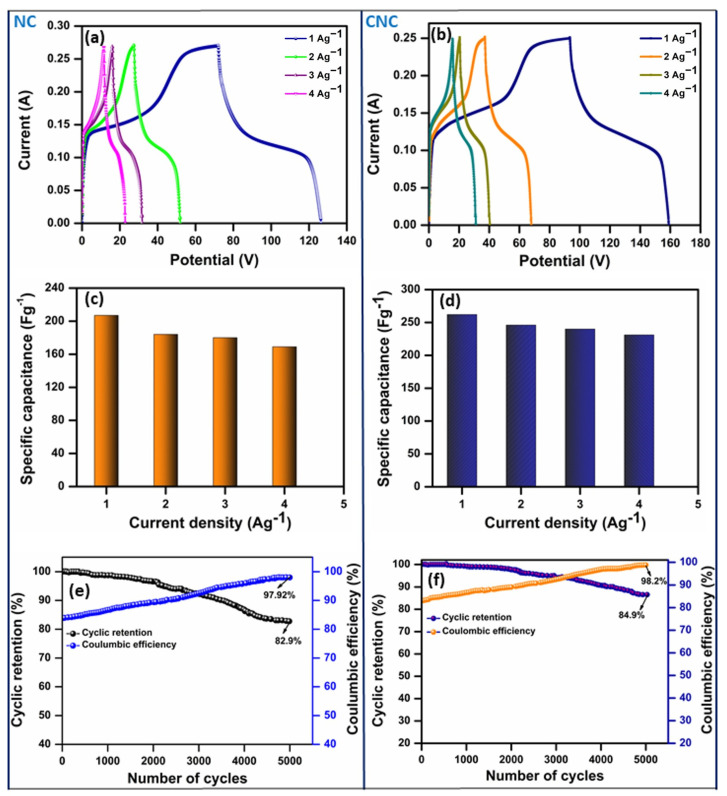
(**a**,**b**) GCD curves, (**c**,**d**) corresponding values of specific capacitance, and (**e**,**f**) cyclic retention and Coulombic efficiency of NC and CNC electrodes.

The galvanostatic charge–discharge analyses of NC and CNC are shown in Figure 9a,b, which investigated various applied current densities ranging from 1–4 mAg^−1^. Non-linear curves were obtained for both electrodes, revealing the pseudocapacitive behaviors and supporting the CV profiles. The specific capacitance of NC and CNC electrodes was estimated using Equation (3).

The calculated specific capacitances are 262, 246, 240, 231, and 228 F/g at an applied current density of 1, 2, 3, and 4 Ag^−1^ for CNC and 207, 184, 180, and 169 F/g, respectively, for NC. The CNC electrode exhibits a greater discharge time at an applied current of 1 mAg^−1^ compared to NC and results in higher specific capacitance. The nanosized morphology with cavities/pores in CNC may enhance the electrochemical performance owing to its increased surface area and greater contribution of ionic conductivity by the Cu^2+^ ions. Additionally, nanorods favored redox reaction kinetics at the interface of the electrode and electrolyte. The variation of the calculated specific capacitances is displayed in Figure 9c,d, which shows the variation of the resultant specific capacitance concerning the scan rates. From the results, it decreased rapidly, which may be caused by the difficulty of accessing the diffusion/intercalation of ions at the interface of the electrode and electrolyte at the higher scan rates. Moreover, the cyclic retention and Coulombic efficiency of the electrode subjected to 5000 cycles are shown in Figure 9e,f. NC has a cyclic retention of 83.5% with a Coulombic efficiency of 98.5%, and CNC demonstrates 84.9% cyclic retention and 98.2% Coulombic efficiency, indicating its remarkable stability as an electrode (Table 1). The OH ions in the KOH electrolyte can easily penetrate the nanoscale pores of the CNC electrode due to the small ionic radius (1.53 Å). Therefore, a longer lifetime was achieved for the CNC electrode than for the NC composite. In addition, electrolytes may be able to restrict the cyclic retention in NC electrodes because they are used in an aqueous electrolyte (KOH) [35]. A similar factor with a lower possibility for the CNC electrode compared to NC is its improved conductivity, which may facilitate the quick movement of ions between the electrode and electrolyte interface. Furthermore, the selection of a desirable potential window is also a key factor in maintaining cyclic stability; it helps control unfavorable Faradaic reactions (detrimental, hydrogen, oxygen evolution) in both electrodes (NC and CNC) [36]. The particle sizes of 89, 112, and 116 nm for Co_3_O_4_, NiO, and CuO have nanoscale effects, allowing their high aspect ratios to construct an interconnected network in the nanocomposite electrode. This may be another factor contributing to obtaining electrochemical features such as high specific capacitance, high-rate capability, and good cyclic retention.

#### 3.4.3. Electrochemical Impedance Spectroscopy Analysis

**Figure 10:**
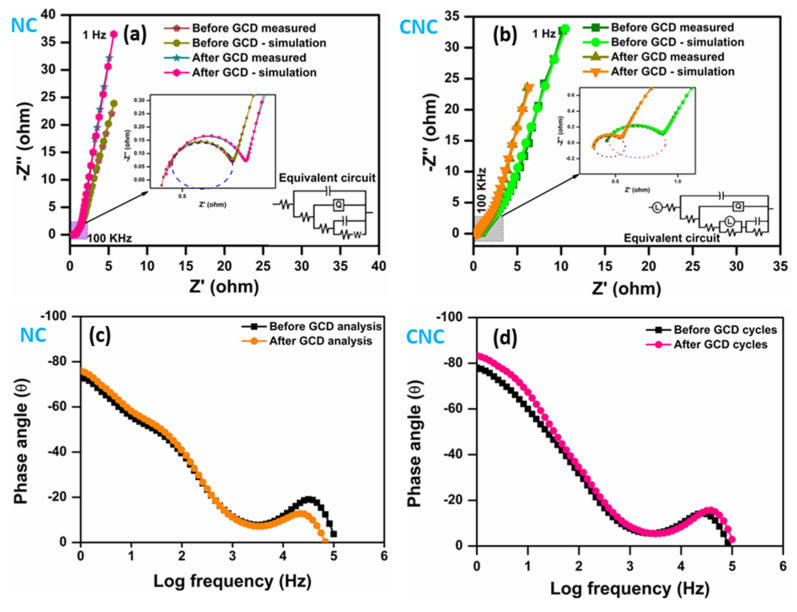
(**a**,**b**) EIS analysis of the prepared NC and CNC composites, (**c**,**d**) corresponding phase angles.

The charge transport kinetics of the NC and CNC composite electrodes were analyzed through the EIS spectra, which are shown in Figure 10a,b. It is formed by two parts: (i) a semicircle in the high-frequency region and (ii) a straight line in the low-frequency region [54]. In the low-frequency region, a parallel combination of resistance and capacitance corresponds to the semicircle, while other diffusion components may cause a straight line in the high-frequency region [34]. A semicircle variation has been found—before and after GCD cycles for both electrodes; their results are shown in the inserted image in Figure 10a,b.

Simulation of the obtained EIS spectra was performed using Z Simpwin software (ZSimpWin_3.60). A charge transfer resistance (R_ct_) and the solution resistance (R_s_) were calculated. The fitted values are summarized in Table 2, and the corresponding equivalent circuit is shown as an inserted image in Figure 10a,b. An equivalent circuit consists of resistances (R_s_) and constant phase elements (CRs), and a diffusion factor (QR) for the NC electrode and an inductance (LR) has been added along with this combination of elements for the CNC electrode. Resistances in the equivalent circuit diagram describe the movement of moving charges that occur between the electrode and the electrolyte surface or vice versa and non-Faradic charges that are modeled as constant phase elements. The inductance (LR) in the CNC electrode describes the metallic conduction in the electrode due to the absorption of Cu^2+^ ions. An estimated (R_ct_) value of 0.27, 0.35 Ω for NC and 0.22, 0.37 Ω for CNC was obtained, which explains the importance of the incorporation of Cu^2+^ ions in the CNC nanocomposite. A low value of (R_ct_) found in the CNC electrode suggested that a more active redox reaction occurred at the interface of the electrode and electrolyte due to the good ionic conductivity. Further, the existence of the mixed valence state of Cu^2+^/Cu^3+^ ions in the nanocomposite of CNC can increase the migration at the electrode interface and lead to greater ionic conductivity. After several cycles of GCD, a small semicircle and the corresponding (R_ct_) values reveal the pseudocapacitive behavior of the CNC electrode. As shown in Figure 10c,d, the phase angle (−83°) is also supported by the obtained pseudocapacitive result of the CNC electrode, which is slightly lower for NC. The phase angle values (−83° and −76°) are close to the value of ideal capacitors (the constant phase angle is −90°). The phase angle of the electrode depends on the frequency of the applied current, the presence of an RC or RCL network, and the pH value of the electrolyte. Acidic and neutral electrolyte media can provide the closest value of −90°, but alkaline electrolyte media provide electrodes/cells with phase angles in the range of ~60 to ~82° [55]. The increased phase angle of CNC electrodes in the KOH electrolyte shows their ability to store charge. Nevertheless, the obtained result is better than the previous reports of Acharya et al., 2021 (−80° for NiO: polystyrene) [56] and Patil et al. 2018 (−68° for Co_3_O_4_@CdS) [57]. Electrochemical studies revealed that nano-sized morphology with pore cavities of CNC nanocomposites and their ionic conductivities were supported for high-performance electrode materials for supercapacitors [58]. The proposed CNC electrodes exhibit excellent electrochemical properties, including specific capacitance, rate capability, cyclic retention, and transport kinetics, as well as material characteristics suitable for supercapacitors to meet real-time applications. Despite the need to surpass the working potential required for electrodes operating with organic electrolytes, this synthesized CNC nanocomposite demonstrates promising capacitive behavior and significant surface areas due to the contributions of Ni [59]. It will be effective for potential consideration in photoconductive applications in the future.

## 4. Conclusions

NC and CNC nanocomposites were successfully produced using the hydrothermal synthesis method. The mixed morphology structures of CNC nanocomposites exhibit enhanced electrode performance. The CNC electrode demonstrated a specific capacitance of 262 Fg^−1^ at 1 Ag^−1^, with a cyclic retention rate of 84.9% after 5000 GCD cycles. Based on the CV profile, this electrochemical analysis aims to explore the energy storage mechanism of electrode materials for supercapacitors. Further, through the design of CNC nano-composite materials, electrochemical stability, electroconductivity, and active surfaces have been achieved. In addition, the importance of metallic conductivity, factors involved in maintaining cyclic retention, and the size and shape of the supercapacitor performance of CNC nanocomposites in aqueous electrolytes were discussed. This discussion may provide guidelines for exploring the potential of mixed metal oxide nanocomposite electrode materials in the electrochemical field with advancements in the future. The hydrothermally synthesized CNC material serves as an electrode, making it a promising contender for applications in supercapacitors. Industries involved in these fields stand to benefit from the convenience of utilizing a single material that is cost-effective and environmentally friendly.

## Figures and Tables

**Table 1:** Electrochemical performance of the CNC electrode compared with recent literature.

Electrode Material	Synthesis Method	Specific Capacitance (F/g)	Electrolyte	Cyclic Stability		Ref.
				Retention (%)	Cycles	
Co_3_O_4_/NiO nanocomposite	Electrochemical deposition	256 (10 mV/s) 233 (20 mV/s)	1 M KOH	87–96	1000	[37]
Fe-CuO	Co-precipitation	170 (0.5 A/g)	1 M Na_2_SO_4_	90.47	5000	[38]
Co-CuO		136 (0.5 A/g)		82.85	5000	
CuO		114 (0.5 A/g)		71.42	5000	
Mn_2_O_3_	Hydrothermal	256.25 (1 A/g)	1 M KOH	90	3000	[39]
Co_3_O_4_ on MWCNT	Hydrothermal	202 (1 A/g)	2 M KOH	-	-	[40]
Co_3_O_4_ nanoparticles	Microwave-assisted	111 (5 mV/s)	2 M NaoH	-	-	[41]
Co_3_O_4_ nanoparticles with surftactant		166 (5 mV/s)		-	-	
C-MnO_x_	Electrospinning	213.7 (0.5 A/g)	6 M KOH	97	1000	[42]
Graphene nanosheet/Co_3_O_4_	Microwave	243.2 (10 mV/s)	6 M KOH	95.6	2000	[43]
Graphene/Co_3_O_4_	Hydrothermal	157.7 (0.1 A/g)	2 M KOH	70	4000	[44]
Graphene/Co_3_O_4_/CoF_2_·4H_2_O	Microwave-assisted hydrothermal	241.8 (1 A/g)	2M KOH	95.2	1000	[45]
Graphene/Co_3_O_4_/CeO_2_	Calcination/Hydrothermal	221 (2 A/g)	1 M Na_2_SO_4_	96	1000	[46]
Honeycomb-like NiO	Hydrothermal	74.4 F/g (0.2 A/g)	6 M KOH	88	2000	[47]
NiO-CNT	Wet chemical	197.7 (1 A/g)	1 M Na_2_SO_4_	91	4000	[48]
CoNi-CNF	Electrospinning	132 (1 A/g)	1 M KOH	85.3	1000	[49]
ZnO/NiO/CuO	Sonochemical	118 (1 A/g)	1 M KOH	89.97	10,000	[50]
MnO_2_@NiO	Hydrothermal	247.7 (0.5 A/g)	1 M KOH	-	-	[51]
MnO_2_-CuO-BaO	Hydrothermal	198 (10 mV/s)	1 M KOH	-	-	[52]
CuO/Co_2_O_4_	Hydrothermal	246 (0.5 A/g)	5 M KOH	89	5000	[53]
**CNC**	**Hydrothermal**	**262** **(1 A/g)**	**1 M KOH**	**84.9**	**5000**	**Present work**

**Table 2:** Simulation parameters for EIS spectra.

Electrodes	R_s_ (Ω)	R_ct_ (Ω)		Q	L (μH)
		Before GCD Cycles	After GCD Cycles		
**NC**	0.44	0.35	0.27	0.0067	-
**CNC**	0.32	0.44	0.22	0.0018	1.13

## Data Availability

The original contributions presented in the study are included in the article/Supplementary Material, further inquiries can be directed to the corresponding author.

## Supplementary Materials

The following supporting information can be downloaded at https://www.mdpi.com/article/10.3390/ma17163976/s1, Figure S1: EDS mapping of the NiO-Co_3_O_4_ overall spectrum (a,b), oxygen (c), cobalt (d), and nickel (e); Figure S2: FTIR analysis of the prepared CuO-NiO-Co_3_O_4_ nanocomposite; Figure S3: UV–Vis absorbance spectrum (a) and optical bandgap (b) of the prepared NiO-Co_3_O_4_ composite; Figure S4: Error bar for electrochemical workstation (CH instrument: model 60008E); Table S1: structural parameters of NC and CNC composites. References [60,61,62,63,64,65,66,67,68] are cited in the Supplementary Materials.
