# Supplementary material for: Investigation of the Electrochemical Behavior of CuO-NiO-Co3O4 Nanocomposites for Enhanced Supercapacitor Applications
Source: Materials (Basel). 2024 Aug 10;17(16):3976. doi: 10.3390/ma17163976 (PMC11355728; doi:10.3390/ma17163976)
Supplement: Supplementary file 1 [file materials-17-03976-s001.zip › materials-3117159-supplementary.pdf]

materials MDPI

Supporting information

Investigation of the Electrochemical Behavior of CuO-NiO-Co_3O_4 Nanocomposites for Enhanced Supercapacitor Applications

Karthik Kannan [1], Karuppaiya Chinnaiah [2], Krishnamoorthy Gurushankar [2,3], Raman Krishnamoorthi [4], Yong-Song Chen [1,*], Paskalis Sahaya Murphin Kumar [5,6] and Yuan-Yao Li [5,6]

Materials

The chemicals $Cu(NO_3)_2.6H_2O$, $Ni(NO_3)_2.6H_2O$, $Co(NO_3)_2.6H_2O$, and NaOH were acquired from Sigma Aldrich in Taiwan with a purity of 99.5%. These materials were utilized in their original state without undergoing any additional purification processes.

Characterization

The synthesized samples were subjected to structural analysis utilizing the XRD (Bruker D8 Advance X-ray diffractometer). The surface characteristics and a chemical composition of the samples were examined through FE-SEM at 10 kV, utilizing a JSM-6500 F instrument from JEOL with EDAX. Functional group analysis of the prepared composite was characterized through VERTEX 70v FT-IR Spectrometer. The optical properties of the composites were assessed using UV-vis spectroscopy with UV2600 Shimadzu spectrophotometer. Electrochemical analyses were conducted using an electrochemical workstation (model-CHI60008E, USA) equipped with a conventional 3-electrode system.

Electrochemical setup

Electrochemical tests were conducted utilizing an electrochemical workstation equipped with a three-electrode configuration [CH Instruments, model: CHI 60008E, USA]. A platinum electrode served as the counter electrode, while a saturated silver chloride (Ag/AgCl) acted as the reference electrode. The working electrode has been prepared to conduct the measurements. Mixed metal oxide composite working electrode was prepared by mixing of metal oxide composite (active material), activated carbon, and polyvinylidene difluoride (binder) in the concern weight ratio of 80:10:10. The obtained mixtures were made into a slurry using N-Methyl-2-pyrrolidone (NMP) as a solvent then coated on Ni foil (1×1) using the Dr. Blade technique. The final product of working electrode was dried at 70°C for overnight. In addition, 1M KOH solution has been used as an electrolyte. CV and galvanostatic charge-discharge cycle analysis were performed in the potential window of 0 to 0.6 V. EIS was also conducted in the frequency range of 1 to 100 KHz.

Table S1. Structural parameters of NC and CNC nanocomposites.

Sample	Phase	Standard lattice parameter (nm)	Calculated lattice parameter (nm)	Crystallite size (nm)
CuO-NiO-Co_3O_4	CuO [1] (monoclinic)	a=0.4692<br>b=0.3481<br>c=0.5156	a=0.4662<br>b=0.3438<br>c=0.5162	41.20
	NiO [2] (cubic)	a=b=c=0.4203	a=b=c=0.4226	21.10
	Co_3O_4 [3] (cubic)	a=b=c=0.4260	a=b=c=0.4232	26.40
NiO-Co_3O_4	NiO [2] (cubic)	a=b=c=0.4203	a=b=c=0.4232	18.90
	Co_3O_4 [3] (cubic)	a=b=c=0.4260	a=b=c=0.4220	28.2

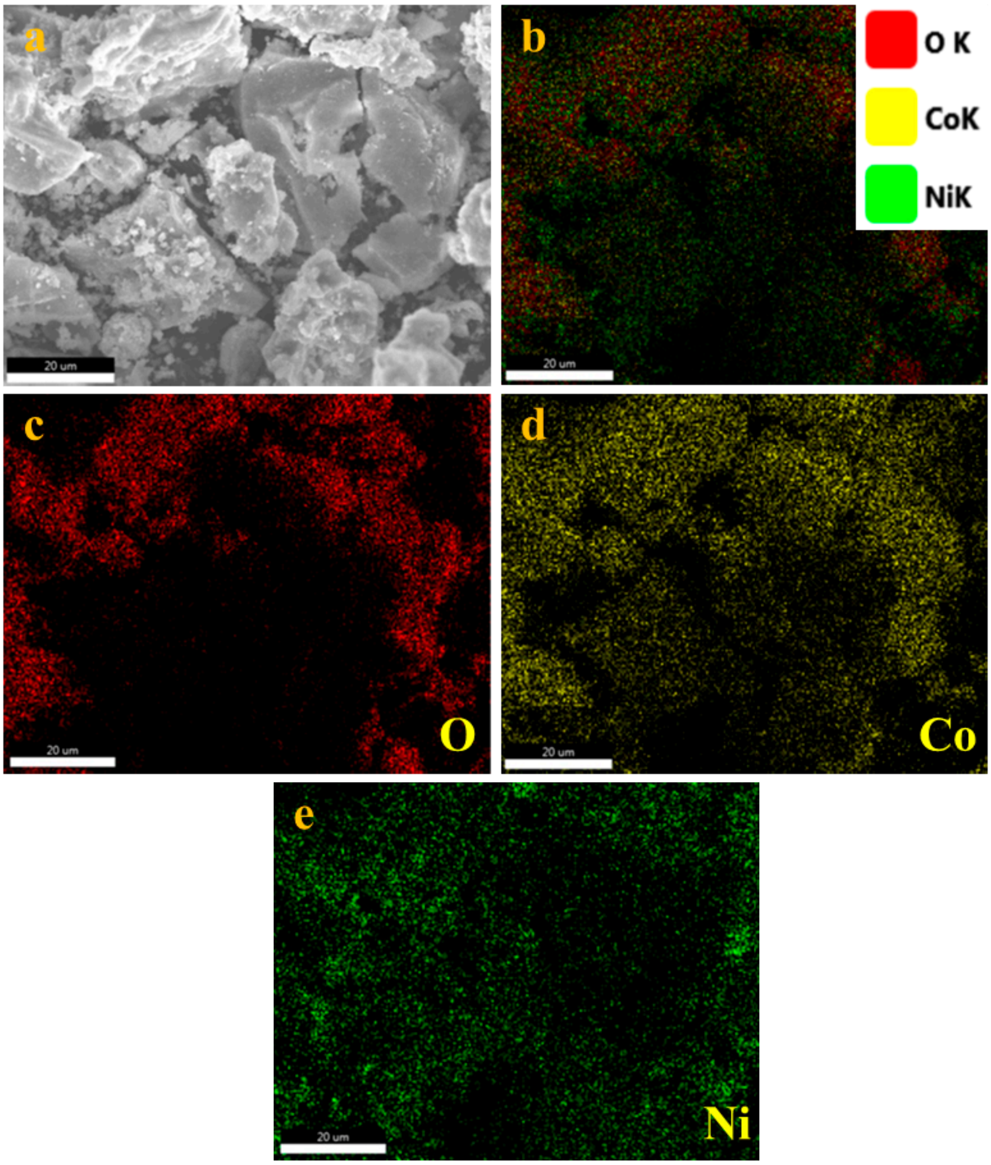

Figure S1. EDS mapping of NiO-Co_3O_4 overall spectrum (a-b), oxygen (c), cobalt (d), and nickel (e), respectively.

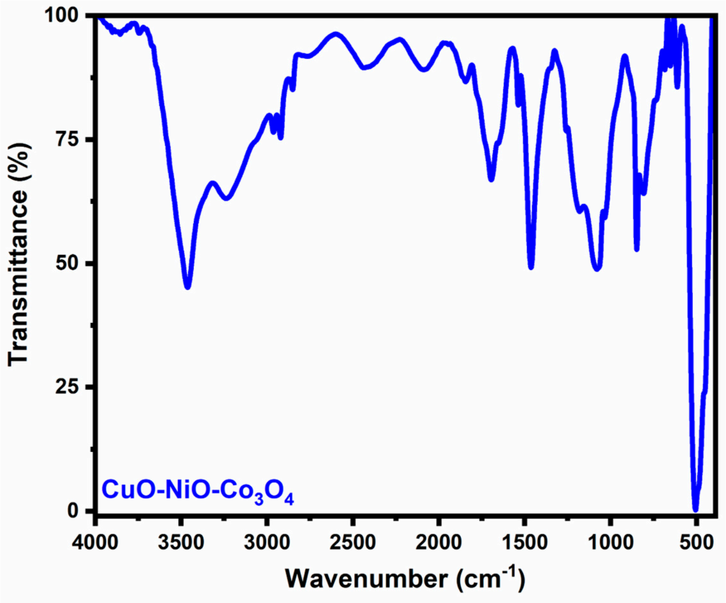

Figure S2. FTIR analysis of CuO-NiO-Co_3O_4 nanocomposite.

FTIR analysis

The FTIR analysis is a useful tool for identifying the presence of functional groups on material surfaces. In Fig. S2 shows the IR spectrum of CNC nanocomposite. The broad-band absorption spectrum at 3467 and 3237 cm^{-1} correspond to the vibration stretching of O–H and a band near 2088, 2447, 2848, 2918, and 2958 cm^{-1} corresponds to the C–H stretching [4]. The band at 1084 cm^{-1} is attributed to C–O stretching. The 1467 cm^{-1} band corresponds to the interacting C–O, while the 1535 cm^{-1} band is indicative of hydrogen-bonded carbonyl stretching and O–C–O stretching of the carbonate on the material's surface [5]. The band at 849 cm^{-1} is attributed to C–O–C bending and supports the interaction with surface ions. The stretching mode of CuO, NiO, and Co_3O_4 is assigned to the peak ranging from 509, 614, 649 cm^{-1} which confirms the formation of metal oxide nanoparticles [6, 7].

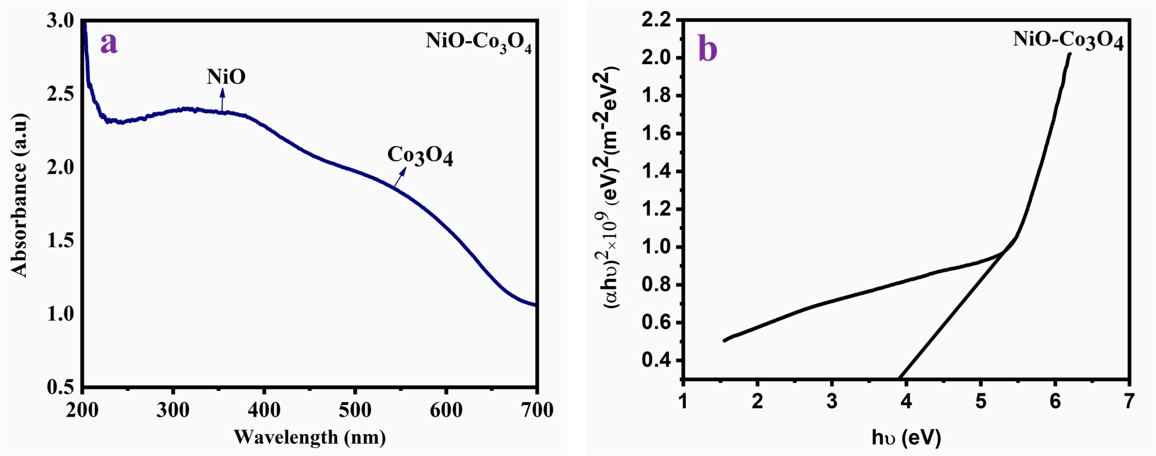

Figure S3. UV-vis absorbance spectrum (a) and optical bandgap (b) of the prepared NiO-Co_3O_4 composite.

Optical analysis

The absorbance and bandgap spectra of hydrothermally prepared NiO-Co_3O_4 composite are displayed in Figure S3. The prepared composite establishment was identified from the absorbance peaks at 354 and 530 nm. The absorbance peak at 354 nm corresponds to NiO and 530 nm is attributed to Co_3O_4. The prepared composite calculated bandgap is 3.88 eV. The wide bandgap of prepared nanocomposite explained the gap between the holes and electrons, which suppressed the recombined activity. The obtained photo exciton charge carriers provoke the oxygen vacancy on the NiO and Co_3O_4 [8]. The charge carrier mitigations and partings may deduce the organic pollutants and deactivate the bacterial system in wastewater and biomedical applications [9].

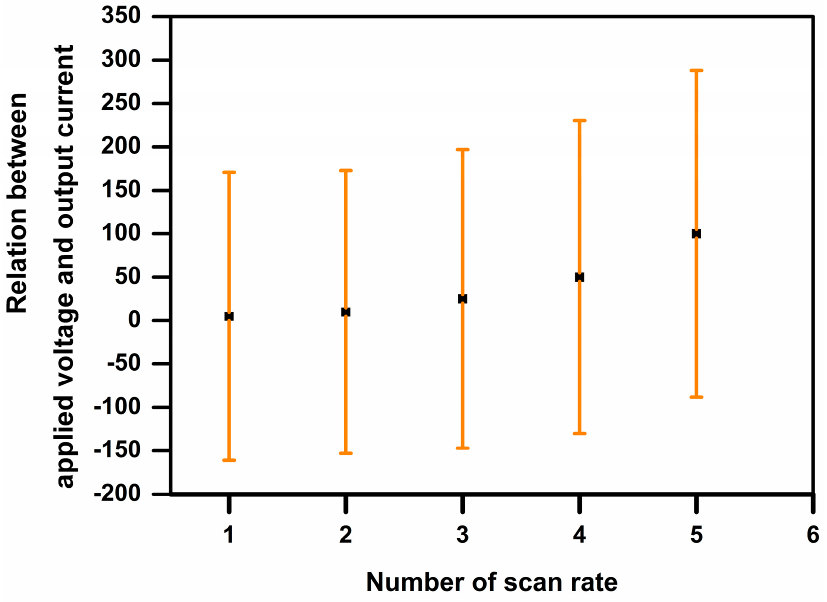

Figure S4. Error bar for electrochemical workstation (CH instrument: model 60008E)

Figure S4 shows the error bars of the data collections depicting the relationship between the applied voltage and output current in cyclic voltammetry. This graphical representation validates the accuracy of the reported measurements. The data collections are almost linear and only start to deviate at high scan rates. Therefore, further evaluation of CV curves was conducted at an optimal scan rate of 25 mVs^{-1}. Consequently, the reported values are highly reliable.

References

1. Mashrafi Bin Mobarak, Md. Sahadat Hossain, Fariha Chowdhury, Samina Ahmed, Synthesis and characterization of CuO nanoparticles utilizing waste fish scale and exploitation of XRD peak profile analysis for approximating the structural parameters, Arabian Journal of Chemistry, 2022, 15 (10), 104117
2. G. Rathika, R. Jagadeeswari, T. Sathiyapriya, P. Selvakumar, Surface and quantum chemical parameters of nickel oxide nanostructures suited for ultraviolet-assisted cationic dye degradation, Kuwait Journal of Science, 2024, 51 (2), 100200.
3. R. Varunamugi, K. Mathu Metha, C. Arun Paul, T. Sathyaseelan, S. Sathiyaraj, T. Prakash, R. Vandamar Poonguzhali, E. Ranjith Kumar, Arunadevi Natarajan, Synthesis and characterization of Co_3O_4, CuO and NiO and nanoparticles: Evaluation of structural, vibrational, morphology and thermal properties, Physics Letters A,2024, 512, 129574.
4. Paul, D., Neogi, S., Synthesis, characterization and a comparative antibacterial study of CuO, NiO and CuO-NiO mixed metal oxide, Mater. Res. Express **2019**, 6 (5), 055004.
5. Maslamani, N., Khan S.B., Danish, E.Y., Bakhsh, E.M., Zakeeruddin, S.M., Asiri, A.M., Super adsorption performance of carboxymethyl cellulose/copper oxide-nickel oxide nanocomposite toward the removal of organic and inorganic pollutants, Environ. Sci. Pollut. Res. **2021,** 28 (29), 38476–38496.
6. Dhiraj Kishor Tatar, Jay Mant Jha, Wet chemical synthesis and characterization of CuO nanoparticles and their application in pool boiling heat transfer, Journal of Crystal Growth, 2023, 617,127305, https://doi.org/10.1016/j.jcrysgro.2023.127305.

7. Chelliah, P.; Wabaidur, S.M.; Sharma, H.P.; Jweeg, M.J.; Majdi, H.S.; AL. Kubaisy, M.M.R.; Iqbal, A.; Lai, W.-C. Green Synthesis and Characterizations of Cobalt Oxide Nanoparticles and Their Coherent Photocatalytic and Antibacterial Investigations. Water 2023, 15, 910. https://doi.org/10.3390/w15050910
8. Feng, Z.; Zhu, X.; Yang, J.; Zhong, K.; Jiang, Z.; Yu, Q.; Song, Y.; Hua, Y.; Li, H.; Xu, H. Inherent Facet-Dominant effect for cobalt oxide nanosheets to enhance photocatalytic CO_2 reduction. *Appl. Surf. Sci.* **2022**, *578*, 151848.
9. Gouasmia, A.; Zouaoui, E.; Mekkaoui, A.A.; Haddad, A.; Bousba, D. Highly efficient photocatalytic degradation of malachite green dye over copper oxide and copper cobaltite photocatalysts under solar or microwave irradiation. *Inorg. Chem. Commun.* **2022**, *145*, 110066.
